# Male-assisted training and injury patterns: hypergraph-enhanced analysis of injuries in women’s water polo

**DOI:** 10.3389/fdgth.2024.1503831

**Published:** 2025-01-06

**Authors:** Xuehui Feng, Zhibin Wang, Zheng Wang, Chen He, Hongxing Xun, Yuanfa Chen, Jie Ding, Gen Chen, Zhe Liu

**Affiliations:** ^1^ Key Laboratory of Sports Trauma and Rehabilitation of General Administration of Sport of the People's Republic of China, Beijing, China; ^2^ National Research Institute of Sports Medicine (NRISM), Beijing, China; ^3^ Hunan Institute of Sports Science, Hunan, China; ^4^ Guangxi Sports Trauma Center, Guangxi, China

**Keywords:** hypergraph, high-order connection, injury patterns, women’s water polo, Male-Assisting-Female-Training

## Abstract

**Introduction:**

The aim of this study is to compare the injury patterns of female water polo players before and after the implementation of the Male-Assisted Female Training (MAFT) program. The study seeks to identify key factors influencing these changes and propose corresponding injury prevention measures.

**Methods:**

We utilized pattern analysis and classification techniques to explore the injury data. A Hypergraph Neural Network (HGNN) was employed for pattern extraction, where each athlete was represented as a node in a hypergraph, with node dimensions capturing high-order relational embedding information. We applied the graph Laplacian operator to aggregate neighborhood features and visualize structural and feature differences in hypergraphs based on different influencing factors. Additionally, we introduced graph structure regularization to improve classification accuracy and prevent overfitting in the relatively small dataset, enhancing our ability to identify critical factors affecting injury types.

**Results:**

The analysis revealed significant differences in injury patterns before and after the MAFT program, with specific influencing factors being identified through both pattern recognition and classification techniques. The classification models, supported by graph structure regularization, achieved improved accuracy in distinguishing key features that contributed to changes in injury types.

**Discussion:**

These findings provide insights into the critical factors influencing injury patterns in female water polo players and highlight the effectiveness of the MAFT program in mitigating certain injury risks. Based on the identified features, we propose targeted preventive measures to reduce injury incidence, particularly in relation to changes brought about by the MAFT training mode. Further research is needed to refine these measures and explore their long-term effectiveness.

## 1 Introduction

Water polo, an aquatic sport that combines swimming, ball skills, and team tactics, has evolved into a global competitive event since its inception in Europe in the late 19th century. The Chinese women’s water polo team, established in 2004, has rapidly progressed from regional competitions to the international stage. Their outstanding performance in international tournaments not only showcases the team’s strength but also reflects the advancement of the national sports sector. In recent years, to further enhance their competitive level, the Chinese women’s water polo team has introduced the Male-Assisted Female Training (MAFT) program. This innovative training method involves sparring with male athletes to simulate higher-level competitive scenarios, thereby enhancing the female athletes’ resilience and tactical execution.

However, the introduction of the MAFT program also presents new challenges, particularly in managing the risk of injuries. Compared to male athletes, female athletes exhibit differences in physical strength and speed, which may increase the risk of injuries during high-intensity sparring sessions. As a team-based combative sport, water polo integrates swimming, throwing, tactical skills, and physical fitness (1). The sport is characterized by intense collisions and grappling in water, lacking the stability of a land environment, leading to frequent injuries during training and matches. Current research on women’s water polo primarily focuses on combat techniques, while injury-related studies are relatively scarce. To date, only one publication has analyzed injuries related to the preparation for the Rio Olympics water polo events (2). Therefore, studying the injury patterns of female water polo players under the MAFT program is crucial for developing effective training plans and injury prevention strategies.

Current research on injuries among elite female water polo players is limited and often focuses on a single body part. Studies have investigated shoulder injuries in elite female water polo players (3), analyzing the incidence of shoulder injuries among players in different positions during matches and the frequency of specific shoulder injury sites. It was found that center forwards and top shooters have the highest rates of shoulder injuries, at 88.89% and 80.95% respectively, with the majority of injuries concentrated in the joints and ligaments . Other research has investigated the impact of water polo throws on the shoulder joint (4), analyzing the effects of throwing actions on injured players, assessing the external rotation stability of injured vs. non-injured players, and providing corresponding recommendations. Lv Zhouxiang and colleagues conducted a study on female water polo players (5), analyzing the multiple injury sites and potential causes, but the study did not delve deeply into the associated movement patterns . The MAFT training model, as a newly proposed strategy, has not yet been subject to authoritative research analysis. Therefore, the research presented in this paper on the injury patterns of female water polo players under the MAFT plan is innovative and crucial for developing effective training programs, reducing injuries during training, and enhancing the performance capabilities of athletes.

The interactions among female athletes in practice are complex and diverse. To better understand and analyze these interaction patterns, this study introduces the concept of hypergraphs. A hypergraph (6–10) is a mathematical model capable of representing complex relationships among multiple nodes. It connects multiple nodes through hyperedges (11–13), which can more accurately simulate the many-to-many interactions among female water polo players. For instance, in a match, not only are there defensive and offensive confrontations between individual players, but the strategic coordination between the entire defense and offense teams can also be represented through hyperedges. This representation method can more comprehensively reflect the tactical layout and collaboration patterns among athletes during matches.

In summary, this study aims to explore the injury patterns, characteristics, and potential coping strategies for female water polo players under the MAFT program. By thoroughly analyzing injury data from training and matches, combined with advanced analytical techniques from hypergraph neural networks (HGNN) (7), we hope to reveal the impact of the MAFT program on the injury risk of female water polo players. The findings will provide scientific evidence for reducing injury incidents, optimizing training methods, and improving athlete performance while ensuring their health and safety. Our contributions are as follows:

1. We analyzed the hypergraph patterns before and after the introduction of the MAFT training program. By comparing the overall hypergraph structure, feature patterns, and the impact of key factors on these structures and patterns, we identified the critical influencing factors associated with the introduction of MAFT.
2. From a classification perspective, we employed graph structure regularization to effectively enhance the accuracy of different injury types in our dataset. This allowed us to more precisely establish an optimized hypergraph structure, thereby identifying the key influencing factors for each injury type before and after the MAFT program.
3. Based on the comprehensive analysis from the aforementioned perspectives, our approach effectively identifies crucial features and subsequently provides recommendations for injury prevention measures.

## 2 Materials and methods

**Figure 1:**
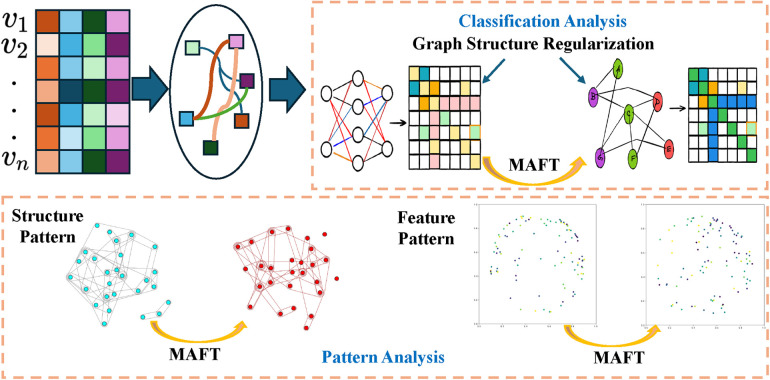
Overview of Hypergraph-enhanced analysis of injury patterns in women’s water polo under Male-Assisting-Female-Training (MAFT) mode.

This study aims to explore the injury patterns, characteristics, and potential coping strategies of female water polo players by comparing injury incidents before and after the implementation of Male-Assisted Female Training (MAFT) and conducting an in-depth analysis of related indicators. To achieve this, we employ the Hypergraph Neural Network (HGNN) as our foundational model to capture the complex relationships and higher-order associative features among athletes, as shown in Figure 1. Subsequently, we perform detailed pattern recognition and factor analysis from a classification perspective to comprehensively analyze the injury characteristics of female water polo players.

The selection of Hypergraph Neural Networks (HGNN) as an analytical method is attributed to its superior capacity in handling complex interrelations and high-dimensional data. In the analysis of injury patterns among elite female water polo players, multiple factors interact and influence each other, such as training load, psychological state, and physiological indicators. Traditional network analysis methods may fail to capture these intricate associations effectively. Firstly, HGNN is a hypergraph-based neural network model capable of managing many-to-many relationships among multiple nodes. This enables HGNN to more comprehensively consider the interplay between various factors when analyzing injury patterns in water polo athletes. Secondly, by constructing hypergraphs, HGNN can visualize the association between different factors and extract key features, aiding researchers in identifying significant factors influencing changes in injury patterns. Lastly, in terms of classification analysis, the study introduces graph structural regularization techniques to enhance classification accuracy and more effectively determine key features that distinguish different types of injuries. The results demonstrate that the rHGNN model, which employs HGNN for classification analysis, exhibits excellent performance in terms of accuracy, positive predictive value, negative predictive value, sensitivity, and specificity. In summary, HGNN, as a network analysis method capable of managing complex interrelations and extracting key features, offers distinct advantages in analyzing injury patterns among water polo players. It assists researchers in gaining a more comprehensive understanding of the factors influencing injury patterns and provides scientific evidence for developing personalized training interventions and injury prevention strategies.

### 2.1 Study design

The study involved 26 athletes from the National Women’s Water Polo Team training between February and July 2021, before and after the implementation of the MAFT mode. Among them, 12 were international-level athletes and 14 were national-level athletes. Their ages ranged from 21 to 32 years, with an average age of 24.9 years. The athletes had been participating in professional sports for 8 to 16 years, averaging 10.9 years, and had been involved in water polo for 8 to 15 years, averaging 9.8 years. They had participated in national-level training for 1 to 14 years, averaging 5.6 years. Twelve athletes had participated in preparation for two Olympic Games. The team positions were distributed as 3 centers, 3 defensive centers, 15 perimeter players, and 5 goalkeepers.

### 2.2 Data collection

In this study, we employed a combination of surveys and clinical diagnoses to analyze and compare the evolution of injury characteristics before and after the implementation of the Male-Assisted Female Training (MAFT) program. The research team spent an extended period residing with the athletes, closely observing the development and progression of injuries. To gather comprehensive data, we used questionnaires to collect basic information, including names, ages, years of athletic experience, injury locations, and causes. Additionally, we recorded pre-analyzed indicators such as training frequency, intensity, duration, recovery time, and physiological metrics like heart rate, blood lactate levels, and VO2 max. The survey also aimed to identify unique injury patterns that emerged following the implementation of the MAFT program. All questionnaires were meticulously collected and processed. During this period, our team was actively involved in diagnosing and treating all athlete injuries, meticulously documenting the occurrence of common injuries and conducting thorough physical examinations. We paid particular attention to any differences in injury processes and severity compared to previous same-gender training scenarios. In summary, through the combination of survey data and clinical records, we captured detailed information on injury locations, types, and severity, providing a comprehensive overview of the impact of the MAFT program on female water polo athletes.

### 2.3 Statistical analysis

Statistical analysis was conducted using SPSS software version 20.0 (SPSS Inc., USA). The proportions of injuries to the neck, shoulder, elbow-wrist, thoracic spine, lumbar-sacral region, hip, knee, ankle, forearm lateral, and sternoclavicular joint were treated as quantitative data. Injury proportions, presented as counts (percentages), were compared between groups using Fisher’s exact test to assess the significance of changes in injury patterns before and after the MAFT mode, with the level of significance set at a two-tailed p-value of 0.05. There was a noticeable increase in the proportion of injuries to joints involved in confrontational activities after the implementation of the Male-Assisting-Female-Training mode. The most common injury site was the shoulder, accounting for 34.6% of injuries, which further increased to 42.3% post-implementation. This was followed by injuries to the lumbar-sacral-hip region, with an increase from 26.9% to 34.6%. Injuries to the elbow-wrist area showed a significant upward trend, rising from 7.7% to 26.9%. Conversely, injuries to the knee and ankle joints, primarily involved in non-confrontational sliding motions, showed a decreasing trend. Notably, two goalkeepers sustained injuries, including bilateral forearm ulnar side hitting injuries and a dominant hand side sternoclavicular joint injury. The details of the sports-related injuries, including the number of cases and the proportion of each injury, are summarized in Table 1. In water polo, the increase in proportional injuries to confrontational joints is particularly notable. Shoulder injuries occur at a rate of 24% to 51%, primarily due to the frequent use of the dominant hand in passing and shooting, as well as the non-dominant hand in defensive actions, leading to bilateral shoulder injuries (14, 15). In confrontational training with male athletes, female athletes need to pass the ball more frequently and attack from various angles, increasing the burden on the dominant hand and raising the injury probability to about 57 Elbow and wrist injuries are also common, with wrist injuries occurring at a rate of 13.6% to 23.1% and elbow injuries at a rate of 6% to 18.2% (16–19). In confrontations with male athletes, female athletes need to find more flexible passing and offensive paths, increasing the burden on the wrist and elbow joints and leading to an increased risk of injury (20, 21). Conversely, the proportion of injuries to stability joints has decreased. The incidence rate of knee and ankle injuries is 4.5% to 10.8% and 6% to 18.2%, respectively (16). Confrontations with male athletes reduce the time spent “treading water,” lowering the tension on the knee joint and thus reducing the injury rate. Among goalkeepers, sternal-clavicular joint injuries are a new type of injury. These injuries are usually caused by direct high-impact trauma, while indirect force-related collateral injuries can be alleviated with rest (22, 23). The inability of athletes to get adequate rest during the preparation period is the main reason for persistent pain.

**Table 1:** This table shows a comparison of injuries to different body parts of female water polo players in traditional training mode and male-assisted Female Training (MAFT) mode. The “Number of Injuries” column in the table shows the frequency of injuries in each part under the two training modes, while the “Injury Proportion” column shows the proportion of injuries in the corresponding part to the total number of injuries. This will provide a key basis for exploring the factors that cause such differences in hypergraph pattern recognition.

Injury	Number of injuries		Injury proportion	
Location	Tradition	MAFT	Tradition	MAFT
Neck	2	1	7.7%	3.8%
Shoulder	9	11	34.6%	42.3%
Elbow & Wrist	2	7	7.7%	26.9%
Chest & Back	1	1	3.8%	3.8%
Lumbar, sacral & hip	7	9	26.9%	34.6%
Knee	11	6	42.3%	23.1%
Ankle	4	3	15.4%	11.5%
Forearm ulnar side	0	1	0%	3.8%
Sternoclavicular joint	0	1	0%	3.8%

### 2.4 Hypergraph construction

A hypergraph is a structure capable of representing complex relationships and multidimensional features, making it suitable for capturing interactions and relationships among athletes. In constructing the hypergraph, we first define nodes and hyperedges.

#### 2.4.1 Node definition

In this study, each athlete is represented as a node $v_{i}$, with a feature vector $x_{i}$ encoding individual attributes such as training intensity, training duration, and injury history. The set of nodes is defined as $V=v_{1},v_{2},\ldots ,v_{n}$, with the node feature vector defined as Equation 1:

(1) $$x_{i}=[\text{Training Intensity},\text{Training Duration},\text{Injury History},\ldots ]$$

The selection of these features is based on their ability to comprehensively reflect the athlete’s condition and performance. Training intensity indicates the effort level during training, where excessive intensity may lead to overtraining and injuries. Accumulated training duration reveals the workload and fatigue accumulation of the athlete, while injury history is a critical indicator for predicting future injury risks. Understanding an athlete’s injury history can help formulate more effective prevention strategies. By incorporating these features, we gain a comprehensive understanding of each athlete’s training status and health condition, providing a solid foundation for subsequent analysis.

#### 2.4.2 Hyperedge definition

In constructing hyperedges, we consider various interactions among athletes, such as passing and defensive actions. These interactions are modeled as hyperedges $e_{j}$, with weights $w_{j}$ representing the frequency and intensity of the interactions. The set of hyperedges is defined as $E=e_{1},e_{2},\ldots ,e_{m}$, where each hyperedge $e_{j}$ connects a group of nodes, indicating interactions among athletes. The rationale behind this choice is that interactions among athletes significantly impact team performance and individual injuries. For instance, frequent passing interactions reflect the coordination and trust among athletes, crucial for both offensive and defensive strategies. In defensive scenarios, cooperation and coordination among athletes are equally important, with the frequency and intensity of defensive interactions revealing the execution and effectiveness of team defensive strategies. Moreover, the choice of hyperedges over simple edges is due to their ability to capture the complexity of multi-party interactions. A hyperedge can connect multiple athletes, representing their cooperation or confrontation within a training unit, whereas simple edges can only represent pairwise relationships, failing to comprehensively reflect the complexity of multi-party interactions. Through this approach, the hypergraph can thoroughly represent the complex relationships among athletes, capturing the interaction patterns and individual contributions within the team, thereby providing richer information for subsequent analysis.

### 2.5 Pattern analysis

To capture the complex relationships within the team, we designed a Hypergraph Neural Network (HGNN) model to learn the embeddings of nodes and hyperedges. The architecture of the HGNN model is designed to capture and represent complex higher-order relationships through a multi-layer structure.

#### 2.5.1 Model architecture

The HGNN model is designed to learn the embeddings of nodes and hyperedges, thereby capturing complex relationships within the team. The embedding function for nodes is defined as Equation 2:

(2) $$z_{i}=\sigma (W^{T}x_{i}+b)$$

where W and b are learnable parameters, and σ is the activation function. This approach maps the multidimensional features of nodes to a high-dimensional space, facilitating subsequent aggregation and analysis. The activation function σ is typically chosen to be a nonlinear function, such as ReLU (Rectified Linear Unit), to introduce nonlinearity and enhance the model’s expressive power.

Specifically, forward propagation is conducted through multiple hypergraph convolution layers to iteratively update the node embeddings:

(3) $$H^{\left(l+1\right)}v=\sigma \left(\sum e\in E\frac{1}{\left|e\right|}\sum_{u\in e}W^{\left(l\right)}H_{u}^{\left(l\right)}+b^{\left(l\right)}\right)$$

In Equation 3, $H_{u}^{\left(l\right)}$ denotes the embedding of node u in layer l, |e| is the number of nodes in hyperedge e, and $W^{\left(l\right)}$ and $b^{\left(l\right)}$ are the learnable parameters for layer l. The activation function σ introduces nonlinearity into the model. This formula updates the node embeddings by aggregating the embeddings of all nodes within the hyperedge, which allows the model to capture the intricate relationships among nodes. In each layer, the node embeddings are refined by aggregating the embeddings of all nodes within their respective hyperedges. This multi-layer structure enables the model to capture higher-order relationships and complex patterns in the data.

#### 2.5.2 Pattern recognition

The hyperedge embedding $a_{j}$ is aggregated as Equation 4:

(4) $$a_{j}=\mathrm{AGG}(z_{i}|v_{i}\in e_{j})$$

where AGG is the aggregation function, typically chosen to be mean or weighted mean. The choice of aggregation function significantly impacts the effectiveness of hyperedge embeddings, as it determines how the embeddings of multiple nodes are combined. Subsequently, a multi-layer perceptron (MLP) in Equation 5 is used to identify injury-related patterns:

(5) $$p_{j}=\mathrm{MLP}(a_{j})$$

During training, we simultaneously train the HGNN and MLP models to minimize the reconstruction error, defined as Equation 6:

(6) $$L=\sum_{\;j=1}^{m}|a_{j}-p_{j}{|}^{2}$$

Through this approach, the HGNN learns efficient representations of nodes and hyperedges, while the MLP identifies injury-related patterns. The minimization of reconstruction error L ensures that the model accurately captures and reconstructs complex higher-order relationships, thereby improving the accuracy of pattern recognition.

By employing the aforementioned methods, we can construct a higher-order hypergraph structure within the overall information and analyze the variation patterns of hypergraph structures under specific influencing factors or indicators. As the final hypergraph is capable of expressing higher-order associations, we can assess the impact of the MAFT program on female water polo athletes’ injuries by comparing the changes in hypergraph structures before and after the implementation of MAFT. Specifically, by evaluating the significance of differences in hypergraph structures and node features, we can identify the key influencing factors.

### 2.6 Classification analysis

We preprocess the data to obtain a feature vector of total dimension $R^{D}(D=320)$, representing the records of all injury-related indicators for the corresponding individual. Then, we use KNN to establish the initial hypergraph structure and utilize the features learned by HGNN for injury type prediction. To better learn the hypergraph structure, we analyzed the characteristics of the dataset and found that introducing a graph regularization mechanism preserves the local geometric structure of the hypergraph data more effectively, preventing overfitting and enhancing the model’s generalization capability. This leads to better classification accuracy, which is beneficial for accurately extracting key influencing factors of injuries before and after the introduction of the MAFT model. Specifically, given an initialized hypergraph structure H(V,E,W), where the symbols denote a set of vertices V, a set of hyperedges E, and a weight matrix W, which is a diagonal matrix representing the weights of the hyperedges. This hypergraph can be succinctly represented by an incidence matrix $H\in R^{\left|V|\times |E\right|}$, where each entry h(v,e) is defined as Equation 7:

(7) $$h(v,e)=\left\{ \begin{array}{cc} 1, & \text{if }v\in e\\ 0, & \text{if }v\notin e, \end{array}\right.$$

Graph regularization in HGNNs aims to preserve the local geometric structure of hypergraph data and improve the model’s generalization capability. This is achieved by adding a regularization term to the loss function that measures the smoothness of node embeddings. Given the H=(V,E,W), the incidence matrix H, the vertex degree matrix by $D_{v}$, and the hyperedge degree matrix by $D_{e}$. The Laplacian matrix L for a hypergraph can be defined as:

$$L=D_{e}-H^{T}WH$$

where $D_{e}$ is the degree matrix of hyperedges, H is the incidence matrix, and W is the weight matrix. During training, we wish for the low-dimensional representations X of the vertices to preserve the local structure of the hypergraph. The regularized objective function for HGNNs can be written as Equation 8:

(8) $$J_{\text{rHGNN}}=\frac{1}{2}X^{T}LX+\lambda \Omega (X)$$

Here, $J_{\text{rHGNN}}$ is the regularized loss function, rHGNN represents the regularized form of HGNN in our proposed model, Ω(X) represents other possible regularization terms (such as weight decay), and λ is the regularization parameter that controls the strength of the regularization term. The optimization process involves minimizing $J_{\text{HGNN}}$ through gradient descent or other optimization algorithms, thereby learning the low-dimensional representations X of the vertices while maintaining the local structure of the hypergraph. This regularization method helps improve the performance of HGNNs on various downstream tasks such as node classification and clustering.

Notably, The regularization term Ω is constructed to ensure that the node embeddings preserve the local structure of the hypergraph. Specifically, Ω(X) includes both a weight decay term that discourages overly complex models and a graph Laplacian smoothness term that encourages nearby nodes in the hypergraph to have similar embeddings. Mathematically, Ω(X) is defined as Equation 9:

(9) $$\Omega (X)=\alpha \cdot \left(\sum_{i,j}W_{ij}\|X_{i}-X_{j}{\|}^{2}\right)+\beta \cdot \left(\sum_{i}\|X_{i}{\|}^{2}\right)$$

where $W_{ij}$ represents the elements of the graph Laplacian L, $X_{i}$ and $X_{j}$ are the embeddings of nodes i and j, respectively. The first term encourages smooth embeddings across the hypergraph, while the second term, weight decay, penalizes large embedding values. α and β are hyperparameters that control the relative importance of the smoothness and weight decay terms, respectively.

## 3 Experiments

### 3.1 Evaluation and metrics

For pattern analysis, we focus primarily on visualization differences. This involves visualizing the structure and features of the hypergraph after optimizing reconstruction loss. We visualize differences in the global hypergraph and the MAFT-induced changes under single-factor influences. Specifically, when visualizing the hypergraph structure, we highlight the clustering of nodes. For visualizing hypergraph features, we employ dimensionality reduction techniques to create 2D visualizations.

For classification analysis, to assess the model’s performance, we utilize several key metrics. Firstly, Accuracy (ACC) is calculated as $\frac{TP+TN}{TP+TN+FP+FN}$, reflecting the model’s overall ability to correctly classify instances across all categories. Sensitivity (SEN), given by $\frac{TP}{TP+FN}$, measures how effectively the model identifies true positive cases. Specificity (SPEC), defined as $\frac{TN}{TN+FP}$, evaluates the model’s capability to accurately recognize true negative cases without misclassifying them as positive. The Positive Predictive Value (PPV), calculated as $\frac{TP}{TP+FP}$, represents the fraction of correctly identified positive cases among all cases predicted as positive. Lastly, the Negative Predictive Value (NPV), given by $\frac{TN}{TN+FN}$, indicates the proportion of true negatives among the cases predicted as negative. These metrics collectively provide a comprehensive evaluation of the model’s classification performance.

### 3.2 Implementation details

For pattern analysis, the MLP consists of two hidden layers with 64 and 32 neurons respectively, followed by a ReLU activation function. The output layer uses a sigmoid activation function to produce the final pattern recognition output. The HGNN and MLP models are trained simultaneously using the Adam optimizer with a learning rate of 0.001. The training process includes early stopping with a patience parameter set to 10 epochs to prevent overfitting. The models are trained for a total of 100 epochs or until the validation loss stops improving.

For classification analysis, the rHGNN model is trained by minimizing the Equation 8. We employ stochastic gradient descent (SGD) as our optimization algorithm, with a learning rate of 0.01 and a decay schedule to adjust the learning rate over time. The training process is carefully monitored to ensure convergence towards a minimum of the loss function. The hyperparameters λ, α and β in the regularization term Ω(X) are crucial for balancing the weight decay and smoothness constraints. The default values are set as λ=0.2, α=0.03, and β=0.005. For more details on the ablation experiments, please refer to the subsequent sections.

### 3.3 Comparison methods

We selected several comparative methods for our evaluation, including MLP, SVM, GNN, B-GNN, HGNN, and HGNNP.

• **MLP** (24): MLP is a class of feedforward artificial neural networks consisting of multiple layers of nodes, each fully connected to the next. It is widely used for classification and regression tasks due to its ability to capture non-linear relationships.
• **SVM** (25): SVM is a supervised learning algorithm that is effective for both classification and regression challenges. It works by finding the optimal hyperplane that maximizes the margin between different classes in the feature space.
• **GNN**: GNNs (26) are designed to perform inference on data described by graphs. They leverage the graph structure to perform node classification, link prediction, and graph classification tasks by aggregating feature information from neighboring nodes.
• **B-GNN** (27): B-GNN is a scalable graph neural network designed to handle large-scale graph data. It introduces techniques to efficiently manage large graphs while maintaining performance, making it suitable for big data applications.
• **HGNN** (7): HGNN extends traditional GNNs to hypergraphs, which can capture higher-order relationships among data points. This method is particularly effective in scenarios where interactions involve more than two entities.
• **HGNNP** (13): HGNNP is an enhanced version of HGNN that includes additional mechanisms to improve its performance. It further refines the ability to capture complex relationships in hypergraph-structured data.

Our proposed method, rHGNN, represents the regularized form of HGNN, incorporating regularization techniques to improve generalization and performance in the context of our specific application.

## 4 Discussion

### 4.1 Study on pattern analysis

**Figure 2:**
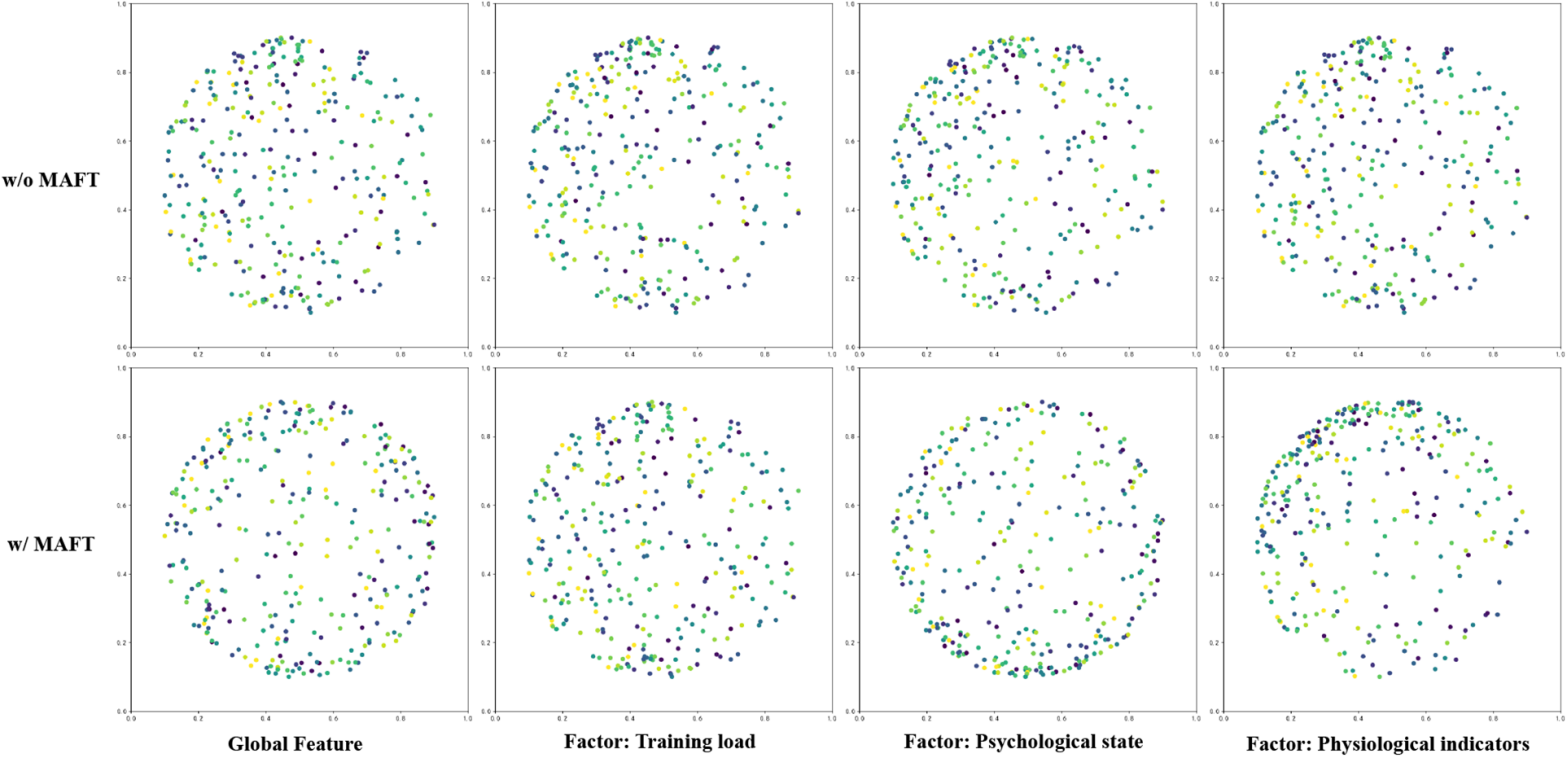
We visualize the positions of all features in the hypergraph within a standard 2D space. By visualizing the feature changes before and after the implementation of the MAFT program, we can observe the most impactful factors. We highlight the three most significant factors: training load, psychological state, and physiological indicators.

Figure 2 illustrates the distribution of various features in a two-dimensional space, comparing the scenarios with and without the application of MAFT. The figure is divided into four subplots, each representing different types of features: Global Feature, Training Load Factor, Psychological State Factor, and Physiological Indicators Factor. In the case of Global Features, it is observed that without MAFT, the data points are sparsely distributed with no apparent clustering pattern. However, when MAFT is applied, the data points become more densely packed and exhibit a certain degree of structural organization. This indicates that MAFT effectively enhances the correlation among global features. For Training Load Factors, a similar trend is observed. Without MAFT, the data points appear scattered and lack discernible patterns. Upon applying MAFT, the points converge into tighter clusters, suggesting that MAFT can extract more meaningful features from training load data. When examining Psychological State Factors, it is evident that without MAFT, the data points are randomly dispersed with no significant clustering. With MAFT applied, small clusters begin to form among the data points. This transformation implies that psychological state data becomes more consistent and interpretable after undergoing feature transformation through MAFT. Lastly, for Physiological Indicators Factors, the untransformed data exhibits a disordered scatter. However, post-MAFT application, the data points reveal clearer structural patterns. This suggests that physiological indicators processed through MAFT better reflect their intrinsic relationships. In summary, the comparison clearly demonstrates that applying MAFT results in more compact and organized distributions across all types of features.

**Figure 3:**
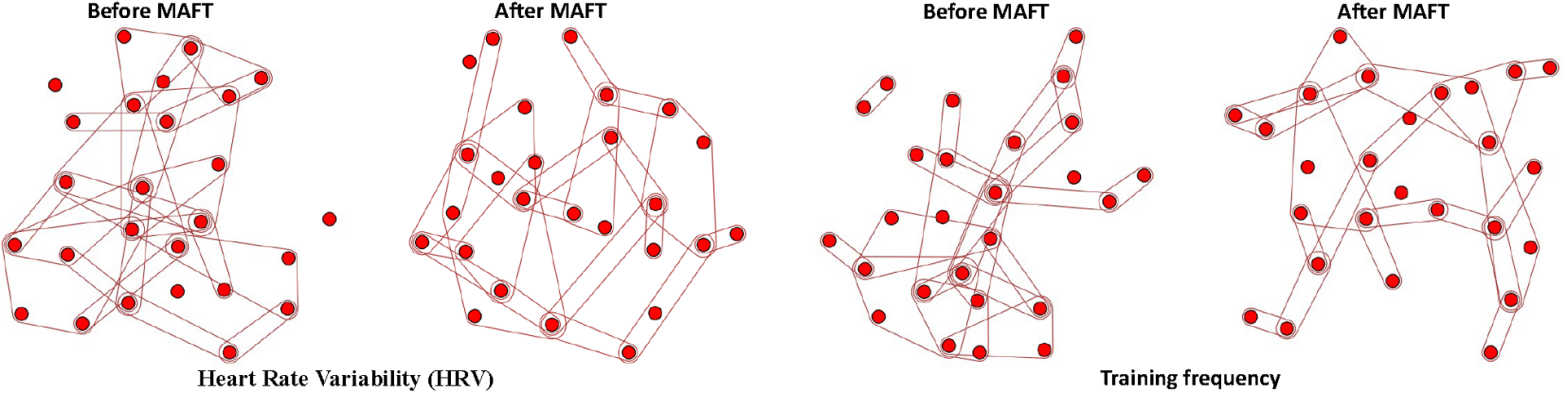
We visualize the changes in hypergraph structures under specific factor indicators before and after the implementation of the MAFT program, highlighting the significant structural differences from the heart rate variability (HRV) and training frequency indicators.

Figure 3 illustrates the changes in hypergraph structures for specific factor indicators before and after implementing the MAFT program, focusing on Heart Rate Variability (HRV) and Training Frequency. Before MAFT, the HRV hypergraph shows numerous dispersed connections with some isolated nodes, indicating weak correlations. This disorganized structure may hinder effective information capture by models. After applying MAFT, the HRV hypergraph becomes more structured and cohesive, with fewer isolated nodes. Enhanced connectivity suggests improved correlation among nodes, facilitating better data utilization by models. Similarly, the pre-MAFT hypergraph for Training Frequency is characterized by scattered connections and weak node associations. This loose network structure can impede meaningful feature extraction. Post-MAFT application reveals a more organized network with tighter clusters and stronger internal associations. This improved structure enhances data consistency and information flow, aiding models in accurately capturing training frequency impacts.

### 4.2 Study on classification analysis

Table 2 presents a comprehensive evaluation of various models, highlighting the performance of our proposed method, rHGNN. The results demonstrate that rHGNN consistently outperforms other methods across all evaluated metrics, including Accuracy (ACC), Positive Predictive Value (PPV), Negative Predictive Value (NPV), Sensitivity (SEN), and Specificity (SPEC). The ACC for rHGNN is notably high at 0.90635, surpassing traditional models such as MLP and SVM, which achieve ACCs of 0.68637 and 0.65817 respectively. This indicates a significant improvement in overall model accuracy. In terms of PPV and NPV, rHGNN achieves values of 0.92572 and 0.91092 respectively, reflecting its superior ability to correctly predict positive and negative cases compared to other models like GNN and B-GNN. Furthermore, the SEN value for rHGNN is the highest among all methods at 0.93216, demonstrating its exceptional sensitivity in identifying true positive cases. Similarly, SPEC is also maximized at 0.92785, indicating robust specificity in distinguishing true negatives. Overall, these results underscore the efficacy of the rHGNN model in achieving superior performance across multiple dimensions compared to conventional approaches such as HGNN and HGNNP. The consistent excellence across all metrics suggests that our method offers a highly reliable solution for the given task, setting a new benchmark for future research endeavors in this domain.

**Table 2:** We compute the accuracy of the proposed method on the testing data, and our method achieves the best results.

Method	ACC	PPV	NPV	SEN	SPEC
MLP	$0.68637_{\pm 0.0625}$	$0.68762_{\pm 0.0594}$	$0.69993_{\pm 0.0627}$	$0.68873_{\pm 0.0571}$	$0.69026_{\pm 0.0608}$
SVM	$0.65817_{\pm 0.0297}$	$0.66373_{\pm 0.0308}$	$0.65843_{\pm 0.0325}$	$0.67367_{\pm 0.0362}$	$0.66267_{\pm 0.0398}$
GNN	$0.78716_{\pm 0.0514}$	$0.77621_{\pm 0.0572}$	$0.76523_{\pm 0.0583}$	$0.77276_{\pm 0.0517}$	$0.75862_{\pm 0.0572}$
B-GNN	$0.82796_{\pm 0.0428}$	$0.82781_{\pm 0.0478}$	$0.81872_{\pm 0.0412}$	$0.82664_{\pm 0.0368}$	$0.81654_{\pm 0.0378}$
HGNN	$0.86621_{\pm 0.0381}$	$0.85163_{\pm 0.0365}$	$0.88245_{\pm 0.0327}$	$0.87833_{\pm 0.0392}$	$0.86924_{\pm 0.0461}$
HGNNP	$0.86273_{\pm 0.0367}$	$0.86252_{\pm 0.0387}$	$0.87326_{\pm 0.0352}$	$0.86237_{\pm 0.0371}$	$0.87944_{\pm 0.0308}$
rHGNN (Ours)	$0.90635_{\pm 0.0288}$	$0.92572_{\pm 0.0286}$	$0.91092_{\pm 0.0349}$	$0.93216_{\pm 0.0365}$	$0.92785_{\pm 0.0367}$

Bold values represent the highest scores.

### 4.3 Study on graph regularization

In Table 3, we provide a detailed and comprehensive analysis of how different regularization hyperparameters affect our accuracy. It is evident that λ is optimal around the 0.1 range, α is best suited for the 0.01 range, and β performs well in the 0.001 range.

**Table 3:** Comparison of model performance under different hyperparameters.

Method	ACC	PPV	NPV	SEN	SPEC
rHGNN (Full)	$0.90635_{\pm 0.0288}$	$0.92572_{\pm 0.0286}$	$0.91092_{\pm 0.0349}$	$0.93216_{\pm 0.0365}$	$0.92785_{\pm 0.0367}$
λ=0.01	$0.89049_{\pm 0.0338}$	$0.90780_{\pm 0.0215}$	$0.89159_{\pm 0.0442}$	$0.91773_{\pm 0.0333}$	$0.91677_{\pm 0.0382}$
λ=0.1	$0.89199_{\pm 0.0233}$	$0.91970_{\pm 0.0240}$	$0.90656_{\pm 0.0416}$	$0.93089_{\pm 0.0348}$	$0.92467_{\pm 0.0351}$
λ=1.0	$0.87687_{\pm 0.0427}$	$0.89560_{\pm 0.0207}$	$0.88212_{\pm 0.0314}$	$0.90082_{\pm 0.0274}$	$0.91050_{\pm 0.0273}$
α=0.001	$0.88735_{\pm 0.0236}$	$0.90289_{\pm 0.0352}$	$0.89358_{\pm 0.0452}$	$0.91173_{\pm 0.0274}$	$0.90539_{\pm 0.0415}$
α=0.01	$0.89949_{\pm 0.0184}$	$0.91696_{\pm 0.0311}$	$0.90673_{\pm 0.0225}$	$0.92826_{\pm 0.0511}$	$0.91930_{\pm 0.0346}$
α=0.1	$0.88597_{\pm 0.0147}$	$0.89671_{\pm 0.0222}$	$0.88850_{\pm 0.0397}$	$0.91942_{\pm 0.0438}$	$0.90754_{\pm 0.0235}$
β=0.0001	$0.89944_{\pm 0.0406}$	$0.91289_{\pm 0.0352}$	$0.90275_{\pm 0.0263}$	$0.92961_{\pm 0.0441}$	$0.90086_{\pm 0.0267}$
β=0.001	$0.88206_{\pm 0.0286}$	$0.90511_{\pm 0.0153}$	$0.90949_{\pm 0.0338}$	$0.92774_{\pm 0.0297}$	$0.91953_{\pm 0.0251}$
β=0.01	$0.88584_{\pm 0.0321}$	$0.89636_{\pm 0.0291}$	$0.90519_{\pm 0.0490}$	$0.92634_{\pm 0.0367}$	$0.91799_{\pm 0.0385}$

Bold values represent the highest scores.

When examining the impact of varying the hyperparameter λ, it is observed that smaller values such as λ=0.01 result in a slight decrease in ACC to 0.89049, while PPV and NPV also show minor reductions compared to the full model. As λ increases to 1.0, there is a more pronounced decline in ACC to 0.87687, indicating that larger values may negatively affect overall accuracy. Adjustments to the hyperparameter α reveal similar trends; for instance, at α=0.001, ACC drops to 0.88735 with corresponding decreases in other metrics such as PPV and NPV compared to the baseline model’s performance. For the hyperparameter β, we observe that at β=0.0001, ACC remains relatively high at 0.89944. However, increasing β leads to lower performance metrics, with β=0.01 resulting in an ACC of 0.88584. Overall, the full model (rHGNN) consistently outperforms variations with different hyperparameters. This suggests that optimal tuning plays a crucial role in achieving superior model performance.

### 4.4 Study on MAFT

The introduction of the MAFT mode has had a significant impact on the injury patterns among female water polo players. HGNN provided valuable insights into the complex interactions within the team and identified specific patterns associated with increased injury risk. These findings suggest that tailored training interventions and injury prevention strategies should be developed, considering the unique demands of the MAFT mode. Future research should focus on validating these findings and exploring additional applications of HGNN in sports injury prevention. We recommend paying attention to the following points when implementing the MAFT training program:

1. It is essential to adjust the frequency of training sessions to prevent overtraining and potential injuries. Sufficient rest periods should be incorporated into the training plan to allow athletes to fully recover. Excessive training frequency can lead to fatigue, decreased performance, and an increased risk of injury.
2. Continuous monitoring of heart rate is crucial to ensure that athletes maintain their heart rate within a safe range during training. Wearable devices can be used for real-time heart rate monitoring, allowing coaches to adjust training intensity as needed. This approach helps prevent cardiovascular strain and optimize performance.
3. Providing psychological support and counseling is vital for helping athletes cope with the stress and challenges of high-intensity training programs like MAFT. Establishing open communication channels enables athletes to express their needs and feedback, fostering a supportive environment.

## 5 Conclusion

This study investigated the impact of the Male-Assisted Female Training (MAFT) program on the injury patterns of female water polo players through hypergraph-based pattern analysis and classification perspectives. We first summarized the overall changes in injuries among female athletes under the MAFT program. Using the collected data, we conducted pattern analysis on the hypergraph structure and features, identified key influencing factors, and proposed enhanced preventive measures within the MAFT framework. Additionally, we analyzed the impact characteristics of various injuries before and after the implementation of MAFT from a classification standpoint, incorporating graph regularization techniques to achieve the highest classification accuracy. Our main findings are as follows:

• The introduction of the MAFT program significantly altered the injury patterns among female water polo players, particularly increasing the proportion of joint injuries involved in confrontational activities.
• Hypergraph Neural Networks (HGNN) provided in-depth insights into the complex interactions within the team and identified specific patterns associated with increased injury risk.
• Our rHGNN model, enhanced by graph regularization techniques, excelled in classification accuracy, positive predictive value, negative predictive value, sensitivity, and specificity, providing reliable scientific evidence for injury prevention.

These findings have important practical implications for water polo training and injury prevention. By adjusting the frequency of MAFT training sessions, continuously monitoring heart rates, and providing psychological support and counseling, tailored training interventions and injury prevention strategies can be developed to meet the unique demands of the MAFT mode. These strategies help optimize training methods, enhance athlete performance, and ensure their health and safety. Future research directions:

• Validate the findings of this study and explore additional applications of HGNN in sports injury prevention.
• Further investigate the impact of the MAFT program on athlete performance and injury risk, especially under varying training intensities and durations.
• Research how to more effectively monitor the physiological and psychological states of athletes through technological means, such as wearable devices, to adjust training plans in real-time.
• Explore the applicability of the MAFT program to athletes of different levels and age groups, and how to adjust training methods based on individual differences.

## Data Availability

The raw data supporting the conclusions of this article will be made available by the authors, without undue reservation.
